# Supplementary figures and images for: Genome-Wide Comparative Analysis Reveals Similar Types of NBS Genes in Hybrid Citrus sinensis Genome and Original Citrus clementine Genome and Provides New Insights into Non-TIR NBS Genes
Source: PLoS One. 2015 Mar 26;10(3):e0121893. doi: 10.1371/journal.pone.0121893 (PMC4374887; doi:10.1371/journal.pone.0121893)

Average number of introns

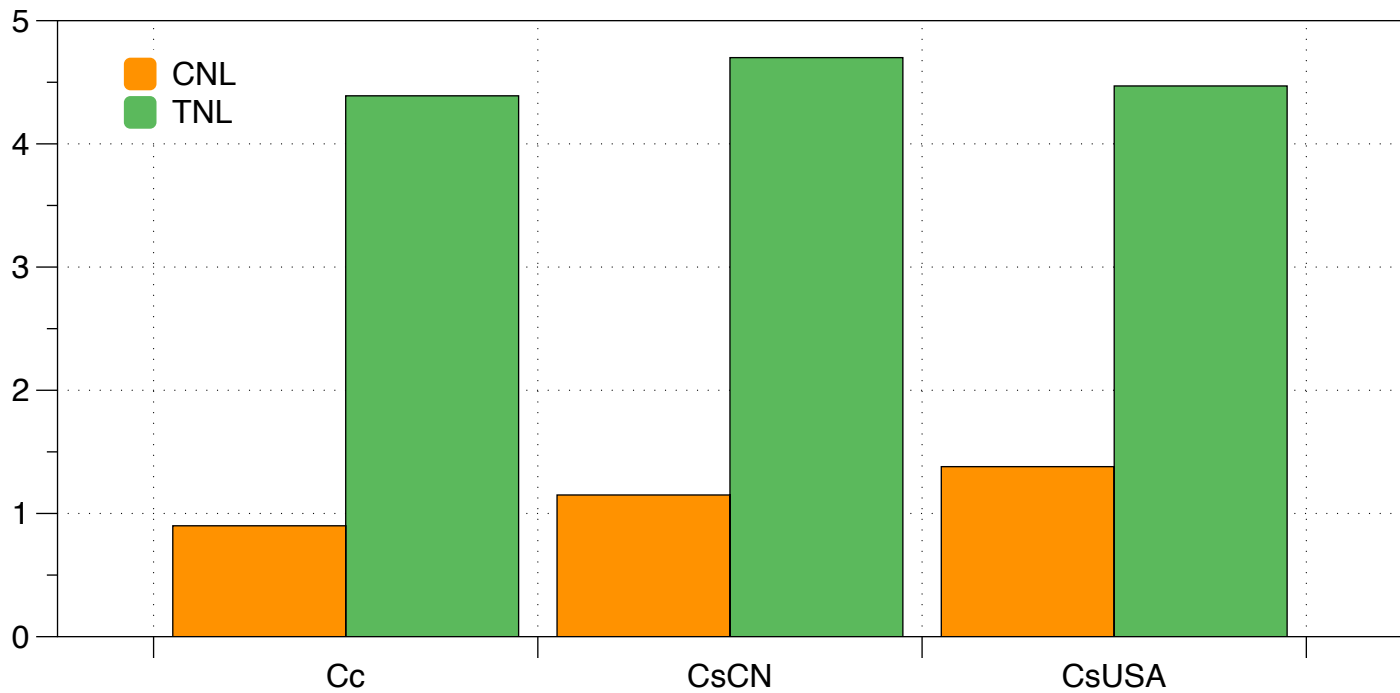

Supplement: S1 Fig — Cc: C. clementina, CsCN: C. sinensis China and CsUSA: C. sinensis USA. (PDF) [file pone.0121893.s001.pdf]

A

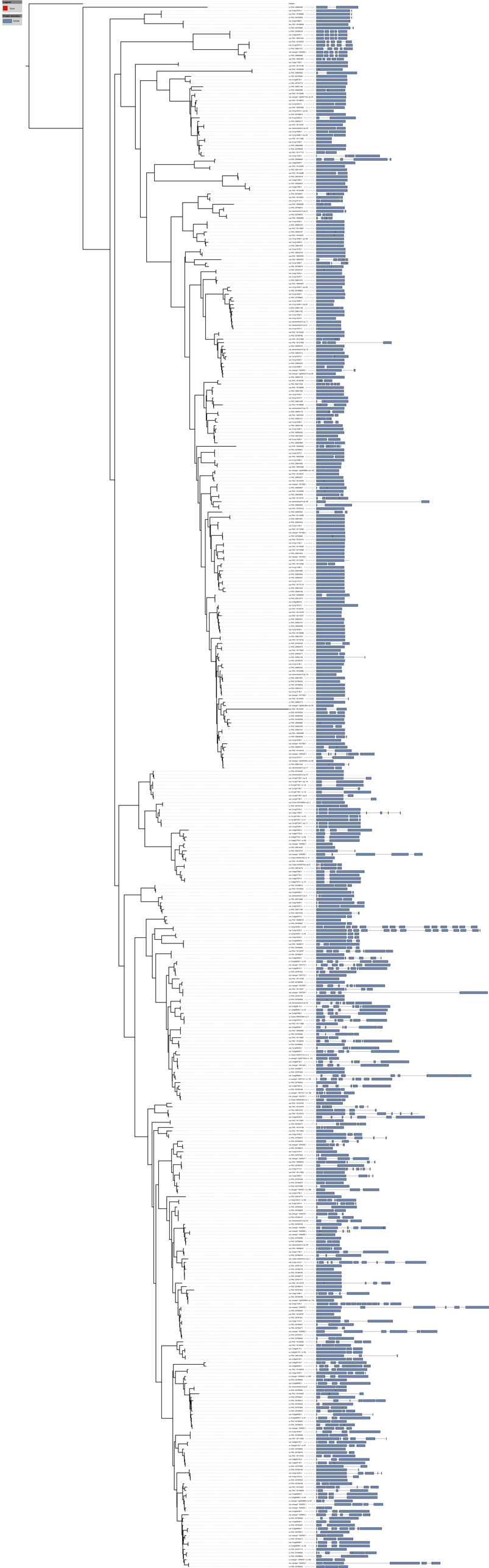

B

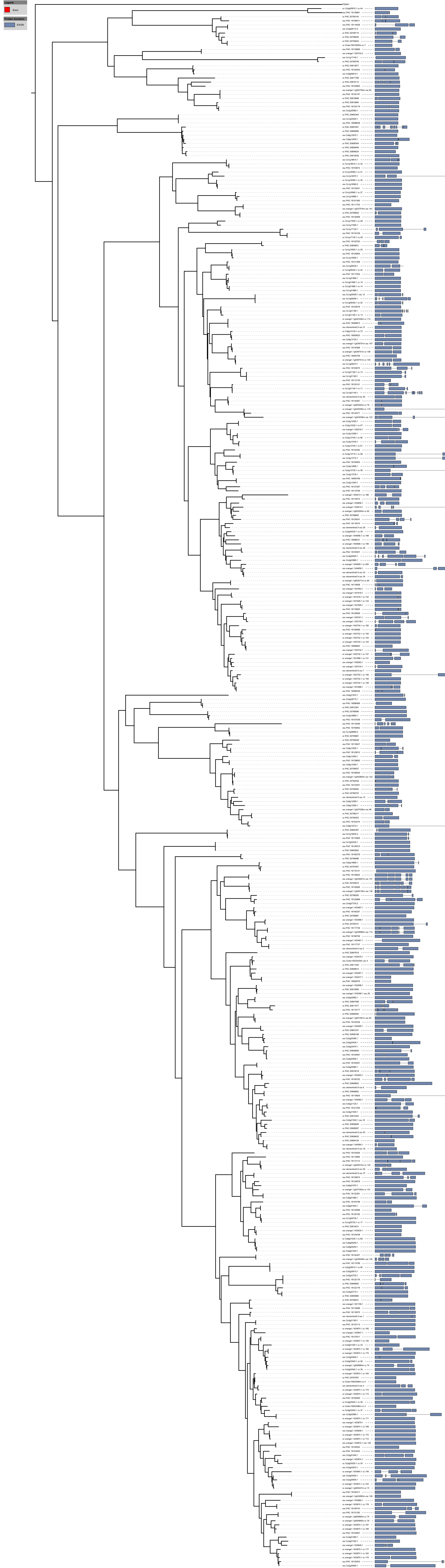

C

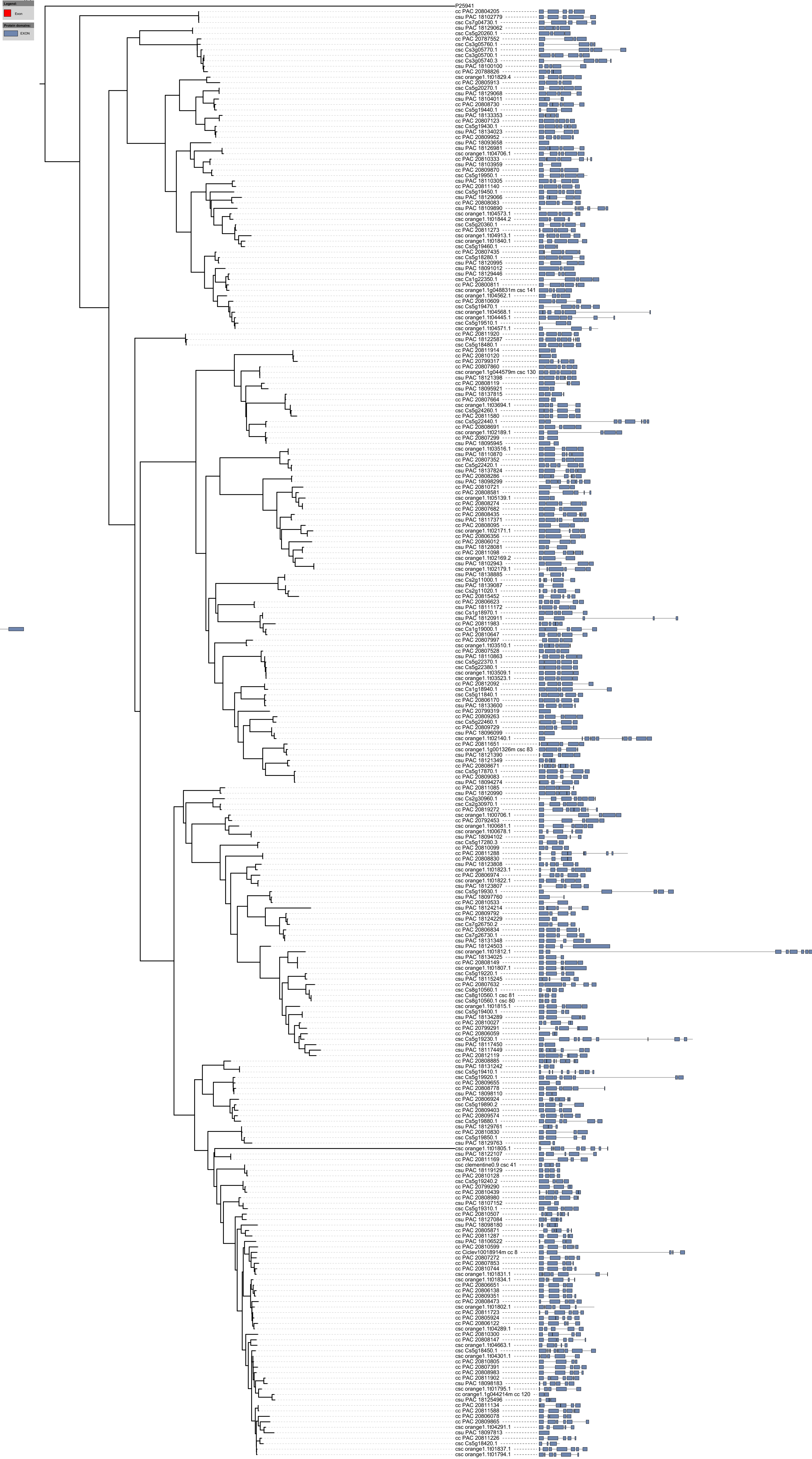

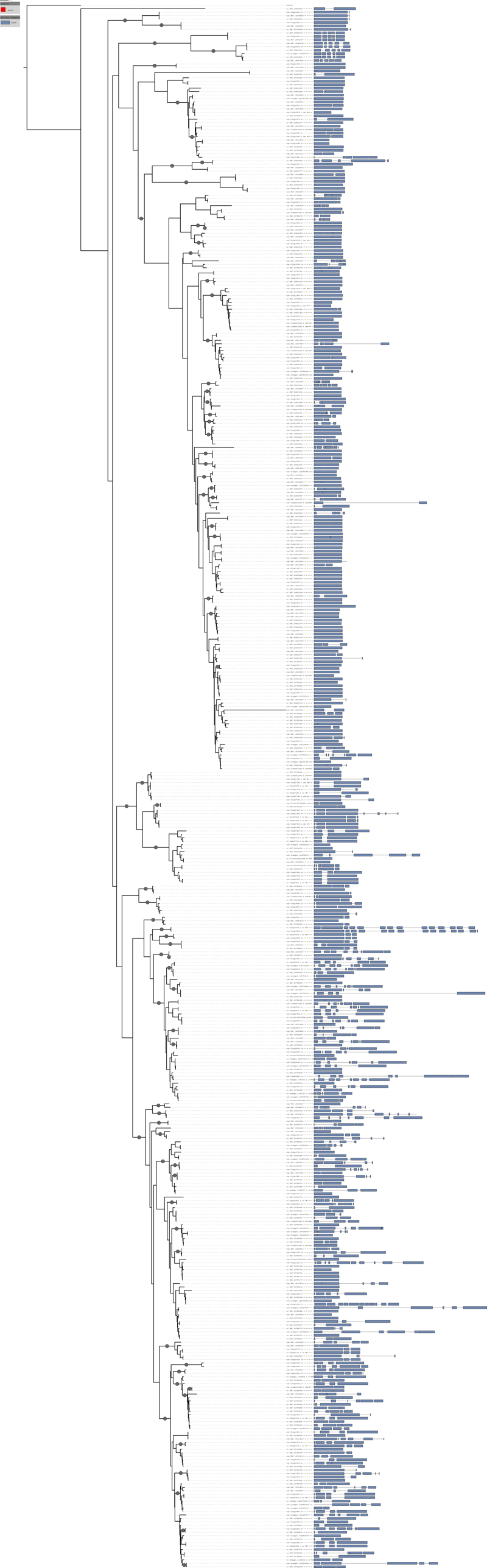

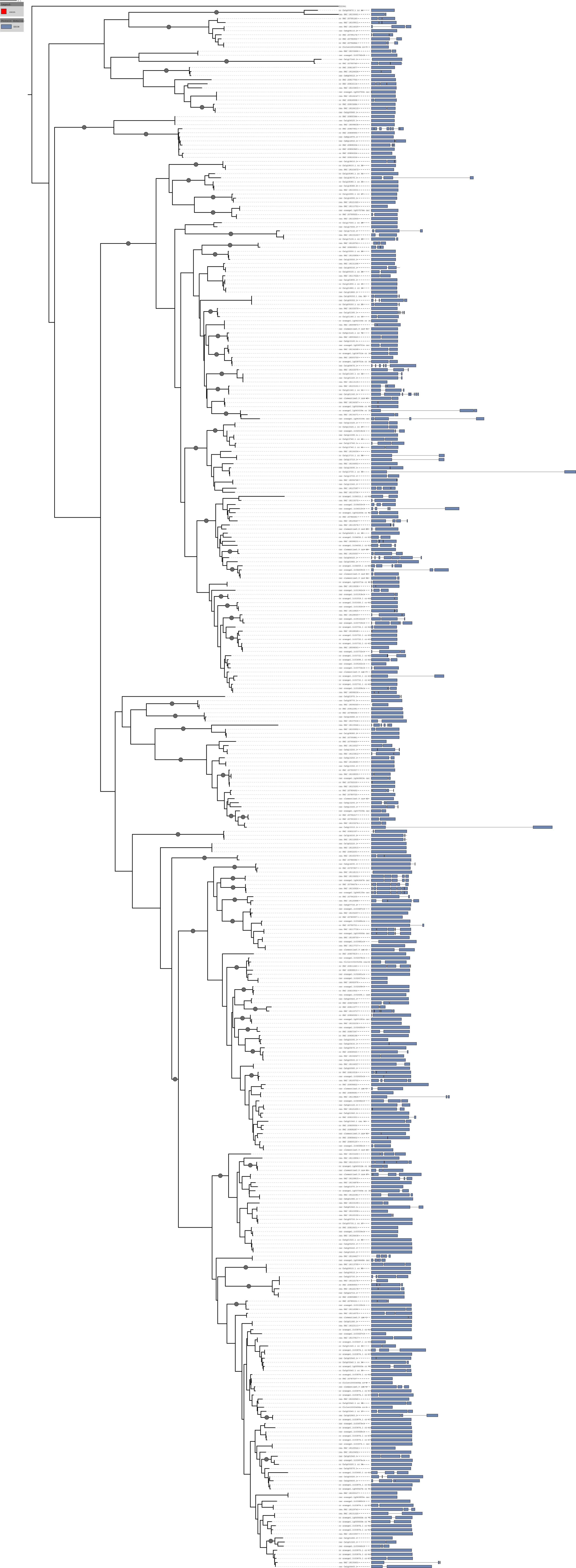

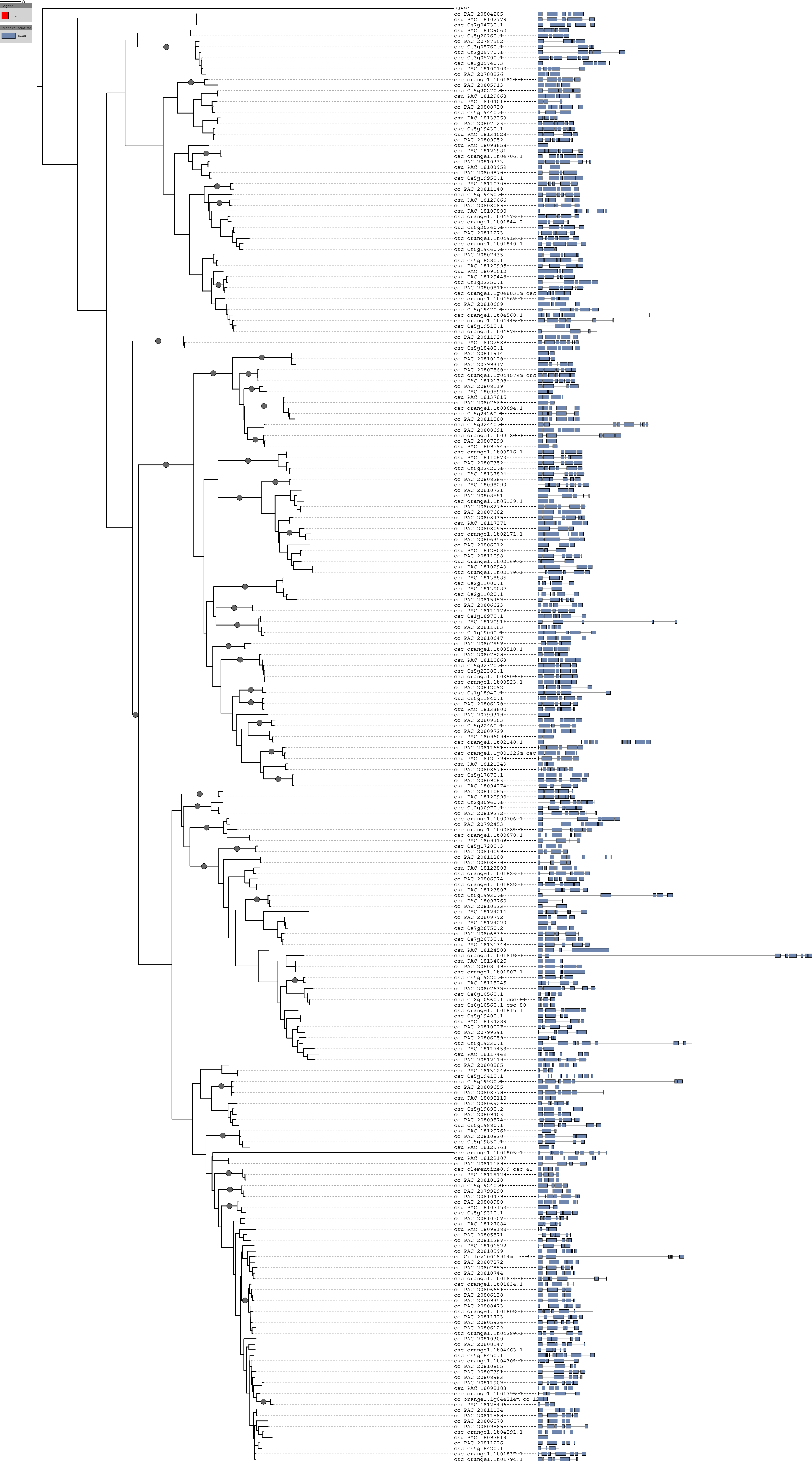

Supplement: S2 Fig — A. CC1 group; B. CC2 group; and C. TIR group. Nodes with bootstrap support of 100 are indicated with grey circles on the tree. (PDF) [file pone.0121893.s002.pdf]

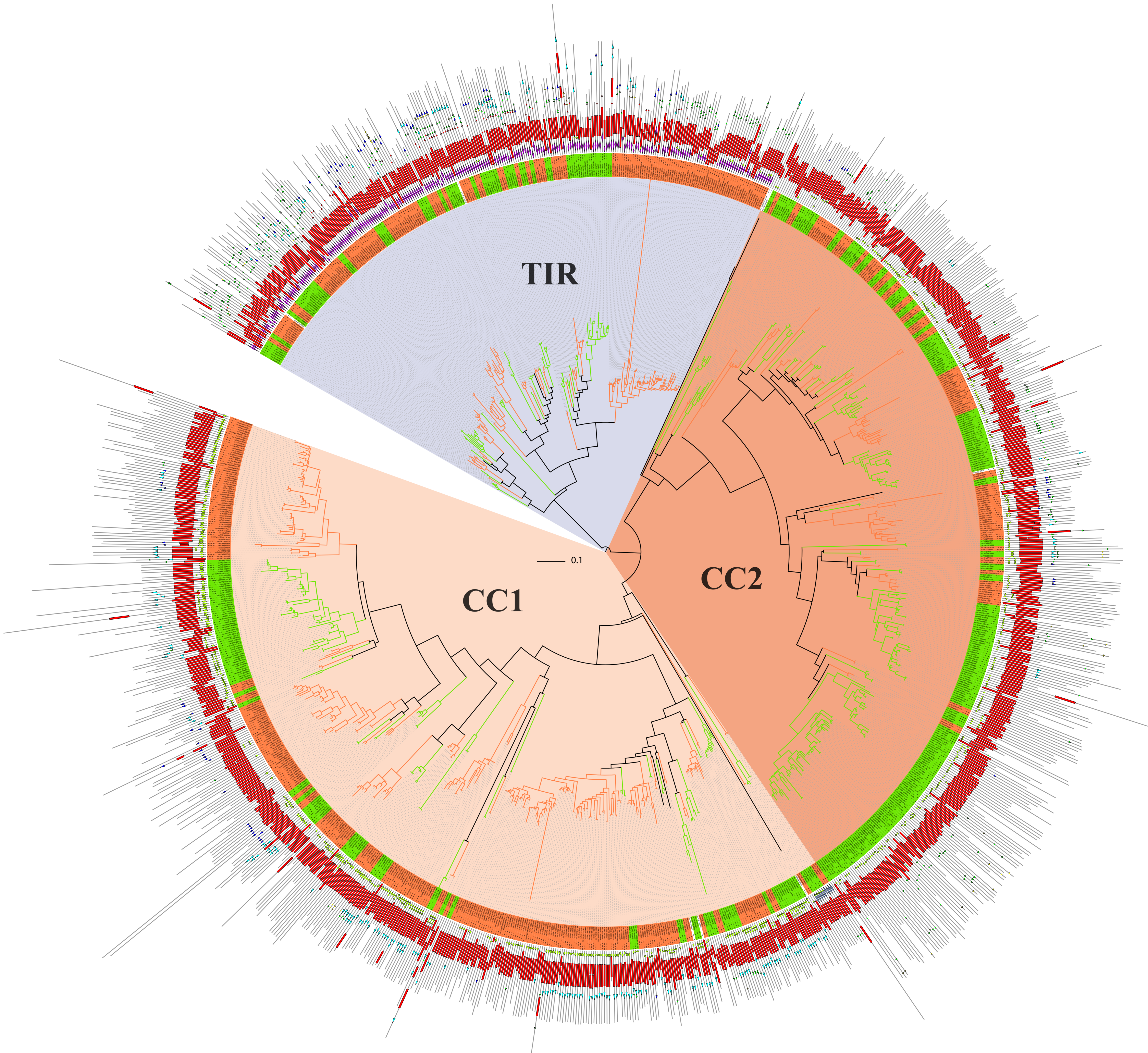

Supplement: S3 Fig — The red rectangles indicate NBS domains, the green ellipses indicate CC domains, the left pointing pentagons indicate TIR domains and the left pointing triangles indicate LRR domains. (PDF) [file pone.0121893.s003.pdf]

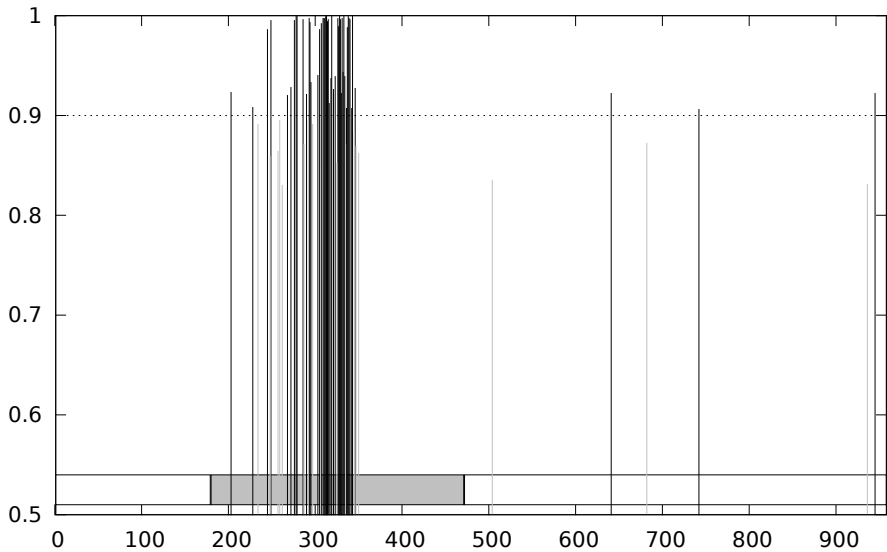

Supplement: S4 Fig — The grey box indicate the NBS domain. (PDF) [file pone.0121893.s004.pdf]

Figure S5

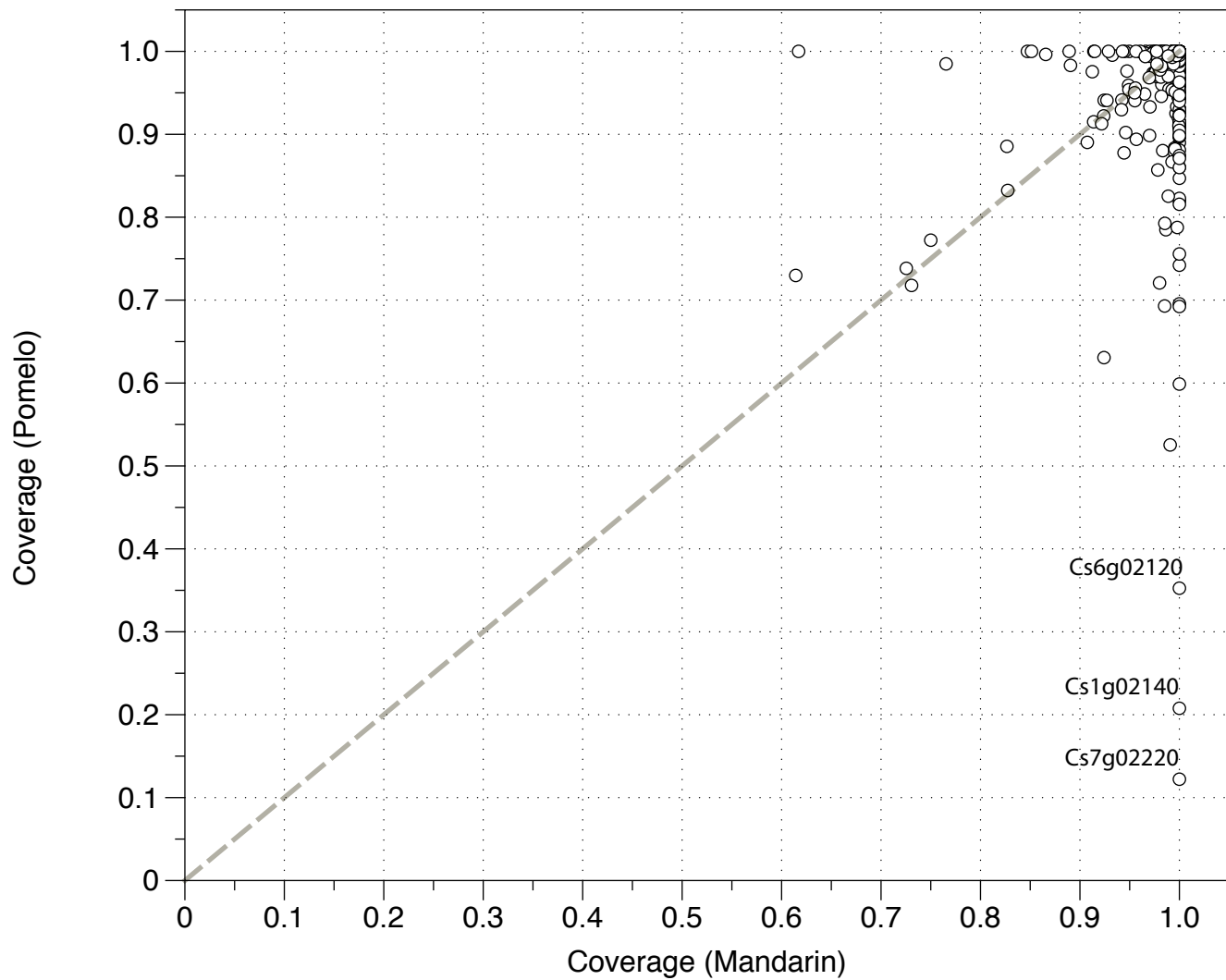

Supplement: S5 Fig — (PDF) [file pone.0121893.s005.pdf]

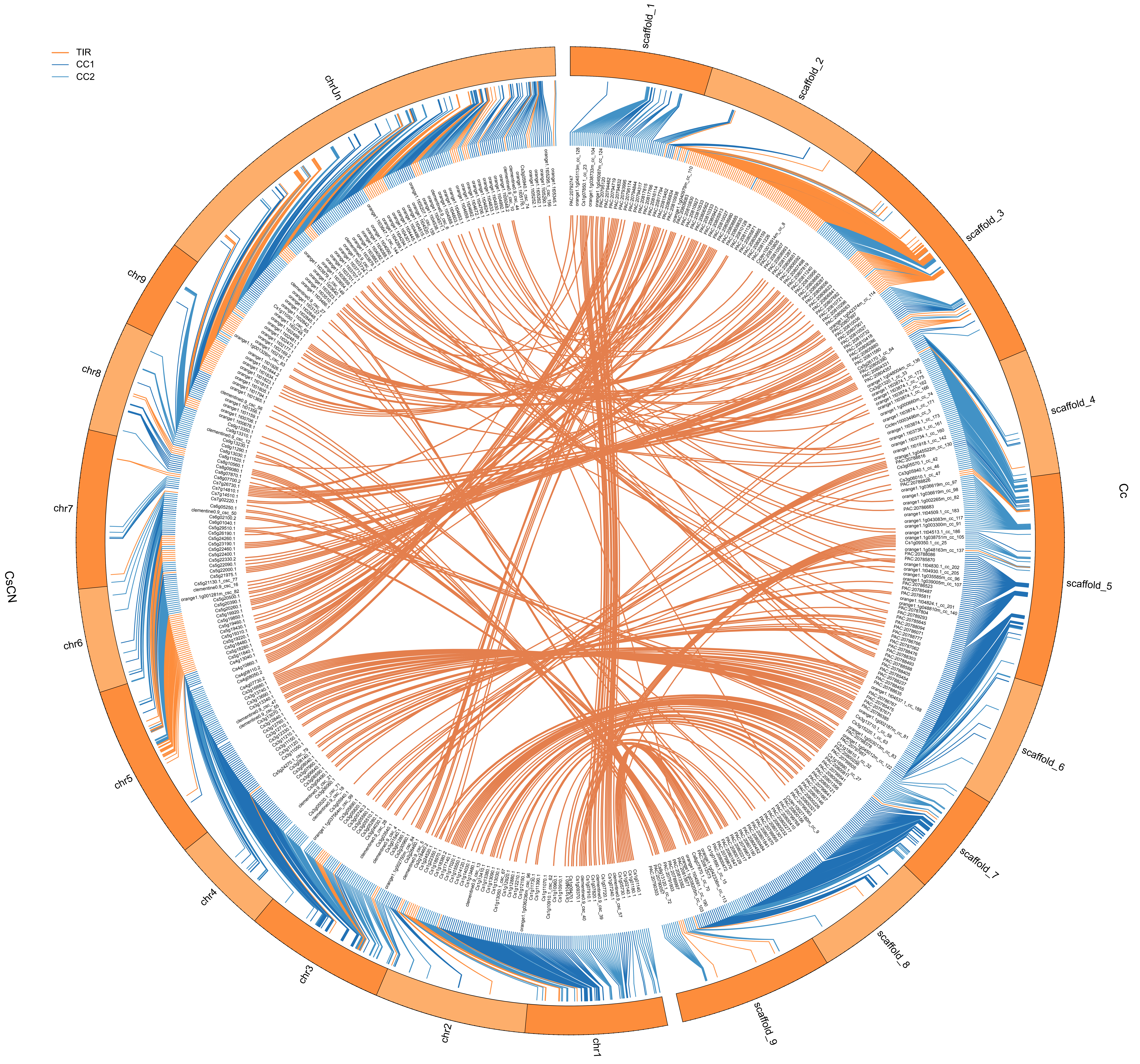

Supplement: S7 Fig — The outer blue circles indicate the nine largest scaffolds of Cc and orange circles indicate nine chromosomes plus a non-localized pseudo-chromosome. The NBS-encoding genes were arranged by their chromosomal positions. The NBS genes in the TIR group are indicated with orange links, CC1 in blue links and CC2 in light blue links. The NBS-encoding orthologs of C. clementina and C. sinensis are indicated by link lines. (PDF) [file pone.0121893.s007.pdf]

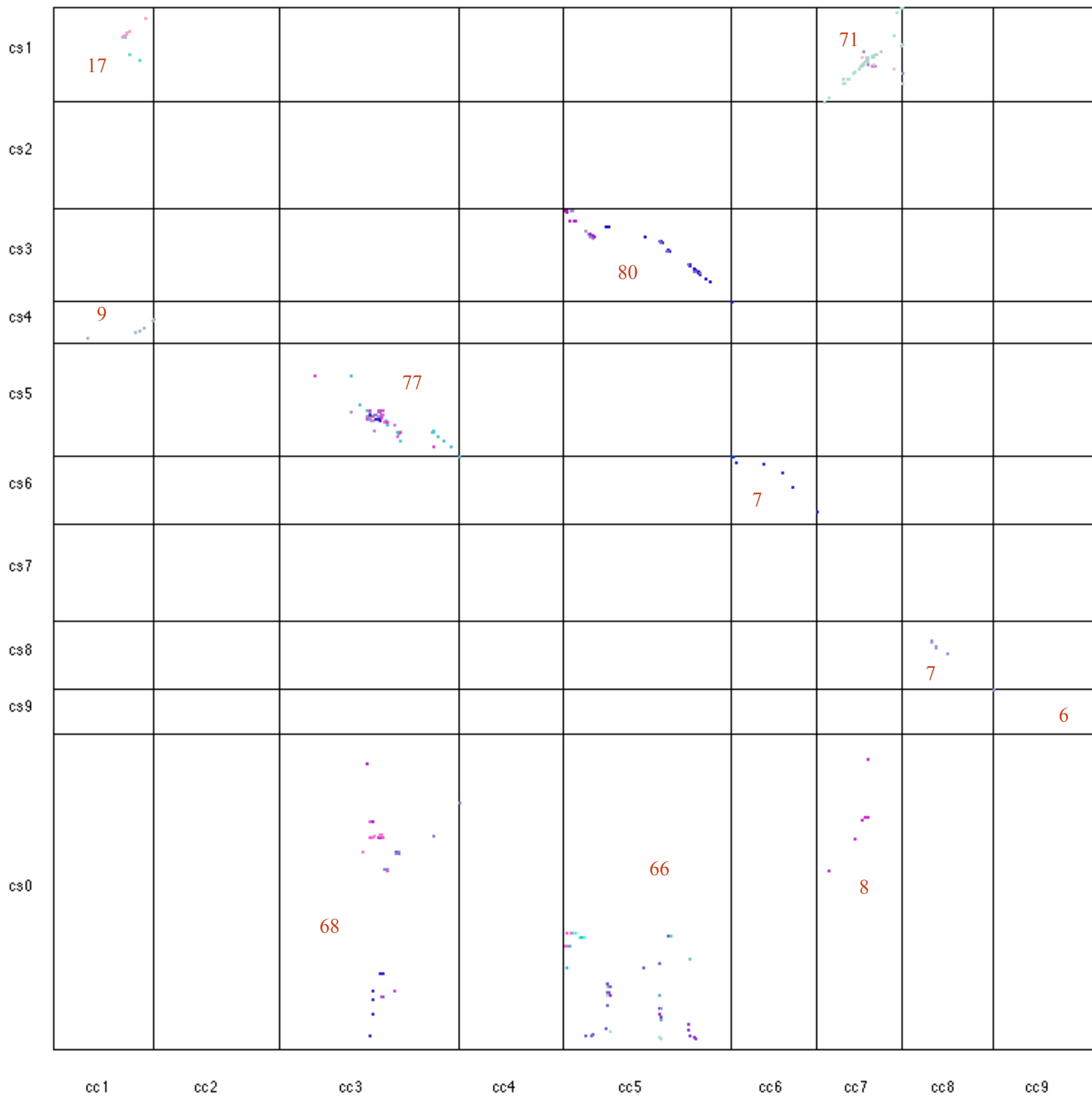

Supplement: S8 Fig — The numbers in the grids indicate the number of syntenic ortholog pairs in the corresponding scaffolds. The scaffolds of C. clementina (Cc) are arranged along the x-axis and that of C. sinensis China (CsCN) are arranged on the y-axis. (PDF) [file pone.0121893.s008.pdf]

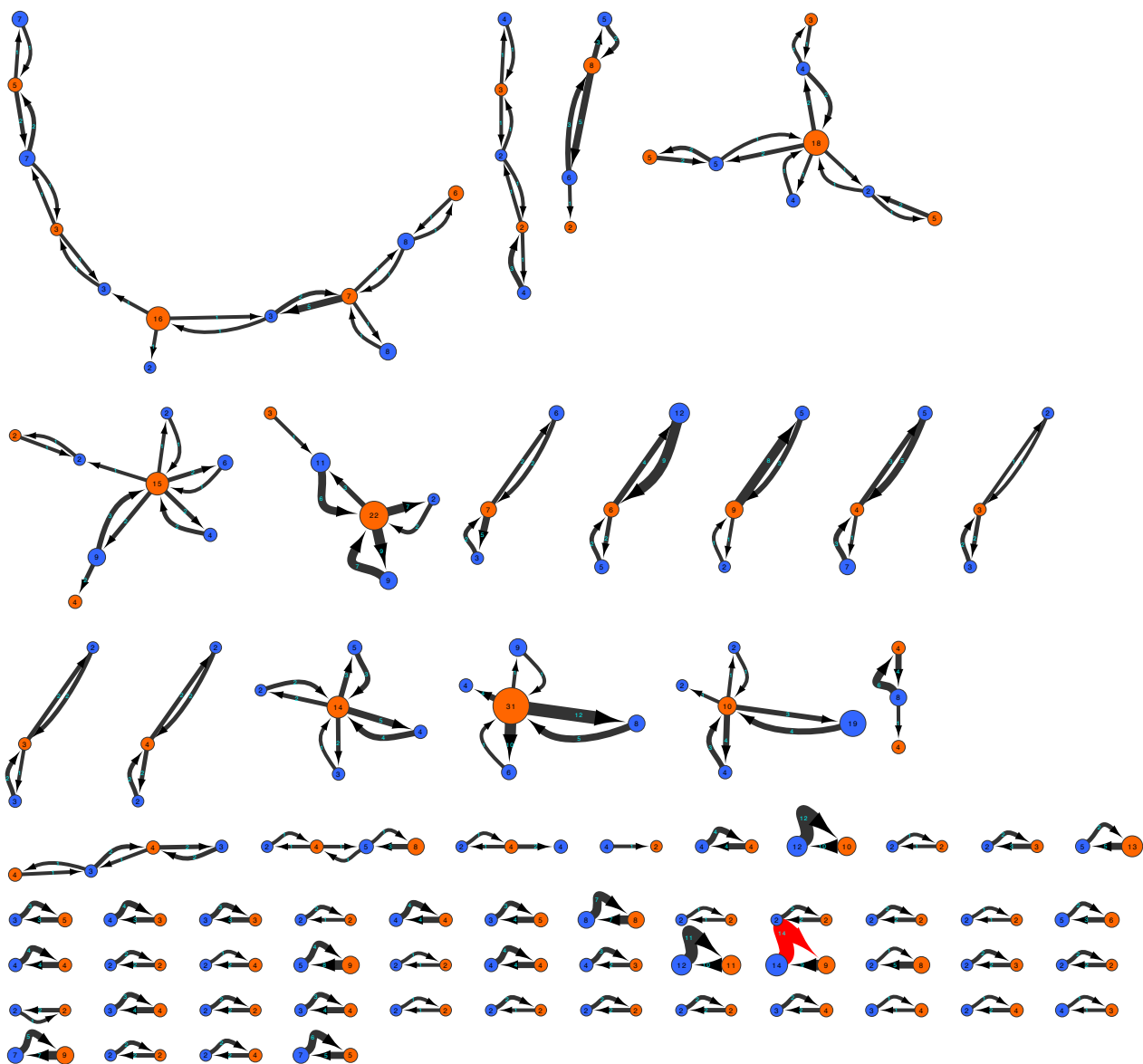

Supplement: S9 Fig — The nodes in orange color indicate clusters of C. clementina, the blue nodes indicate clusters of C. sinensis, and the sizes of the nodes are shown relative to cluster size. The numbers on the edges indicate the number of orthologs identified in other species. (PDF) [file pone.0121893.s009.pdf]

## LRR domains

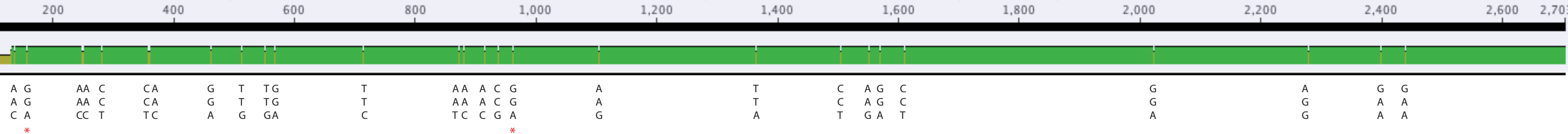

orange1.1g043039m

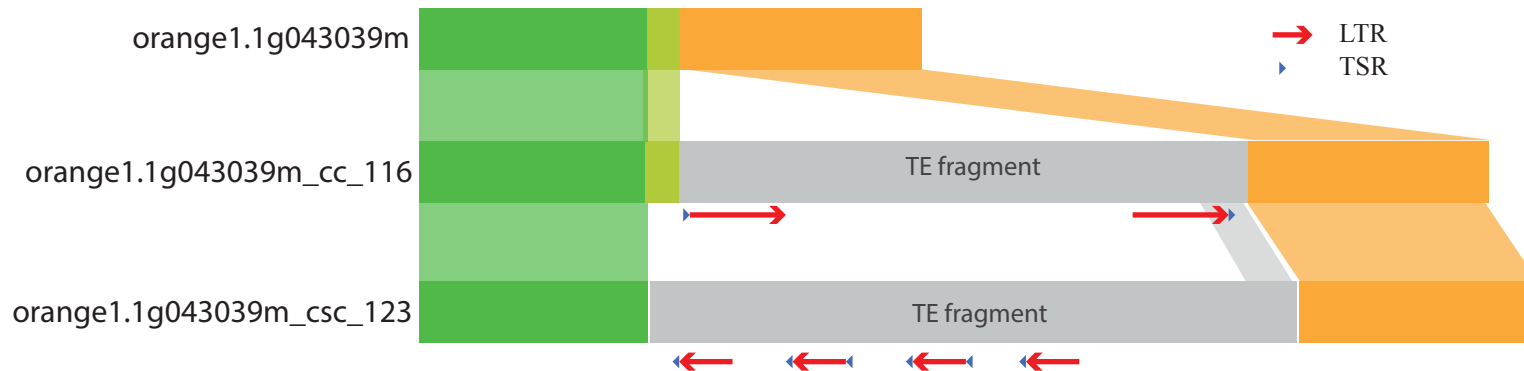

Supplement: S10 Fig — A: Sequences alignment of Cs1g18610.1, Cs1g18610.1_cc_32 and orange1.1g003367m. The bases indicated the variation bases in each Citrus species. The star in red color represented stop-codon gaining mutation and may result in pseudogene of Cs1g18610.1. B: LTR retrotransposon insertion in NBS-encoding genes orange1.1g043039m_cc_116 and orange1.1g043039m_csc_123 from C. clementina and C. sinensis respectively, and the multiple sequences alignment of the orthologs. LTR, Long terminal repeats; TSR, Target site repeats. (PDF) [file pone.0121893.s010.pdf]

A

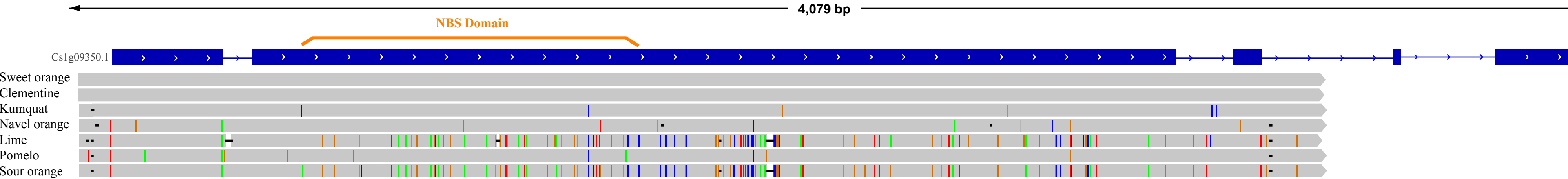

B

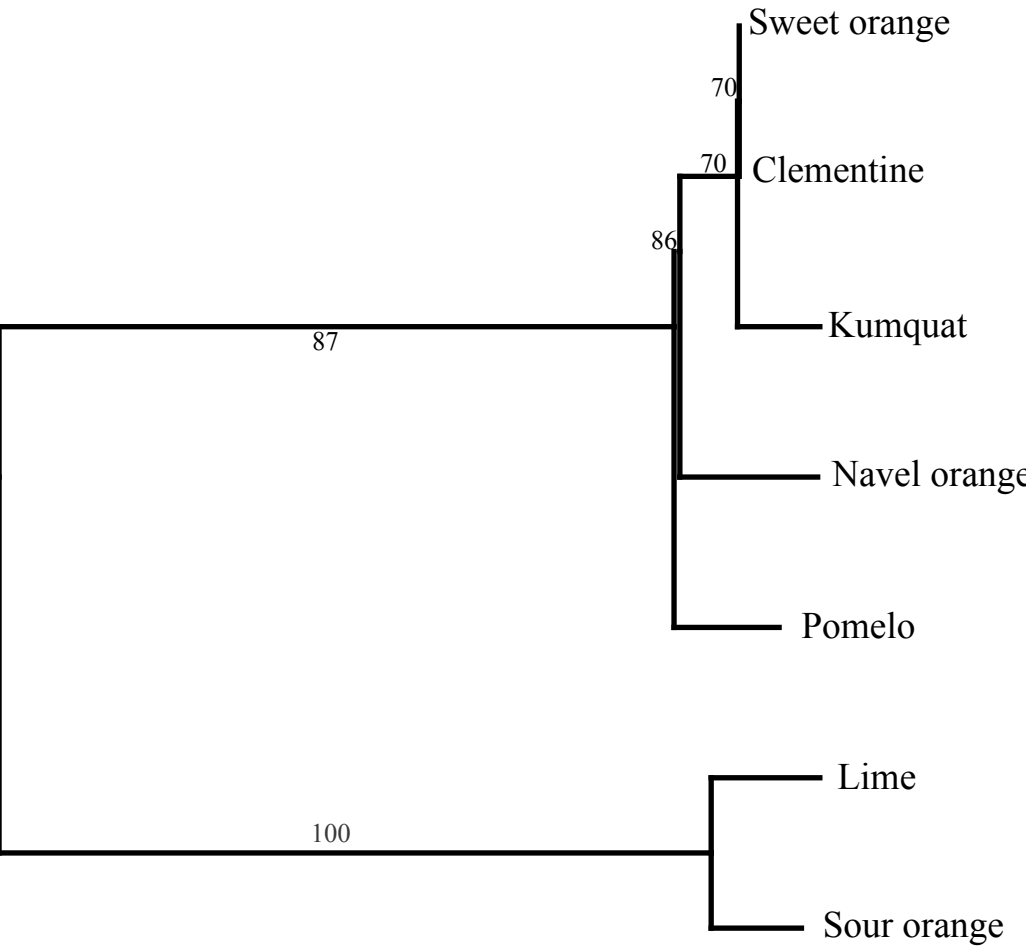

Supplement: S11 Fig — A) multiple sequence alignment, with blue bars along exons and thin lines along introns. The vertical lines in each alignment blocks indicate mutations that differ from the reference sequences of C. sinensis. B) neighbor-joining phylogenetic tree of the orthologs of Cs1g09350.1 in different Citrus species and bootstrap support displayed on the branches. (PDF) [file pone.0121893.s011.pdf]
