# Supplementary material for: Genome-Wide Comparative Analysis Reveals Similar Types of NBS Genes in Hybrid Citrus sinensis Genome and Original Citrus clementine Genome and Provides New Insights into Non-TIR NBS Genes
Source: PLoS One. 2015 Mar 26;10(3):e0121893. doi: 10.1371/journal.pone.0121893 (PMC4374887; doi:10.1371/journal.pone.0121893)
Supplement: S6 Fig — (PDF) [file pone.0121893.s006.pdf]

A

```
Citrus_CC1-1          GSQEIDKLCLGGYCSKNCKSSYKFGKQVA--------------- 29
Arabidopsis_CC1-1     ---EIQRLCLCGYCSKNCCSSYRYGKRVFLMLEEVEKLKSQGFF 41
                         **::*** ******* ***::**:*

Citrus_CC1-2          EEQQQMRRLNQVQGWLSRVEA-------- 21
Arabidopsis_CC1-2            LAQVQVWLSRVQTIENQFNDL 21
                             * *** *****::
```

B

```
Citrus_CC2            -----------------------AVKHWLGKLQDAAYDVEDVLDEWQTEAFR 29
Rice_CC2              -------------------------KXWLXELRELAYDAEDCIDEF------ 21
Arabidopsis_CC2       LKTELTCIHCYLKDAEARQREDEVVKHWVAGIRDAAYDAEDILDTYFLKA-- 50
Populus_CC2           -------------DAEEKQWTNEAVKDWLDDLKDAAYDADDV---------- 29
                                                 * *:   ::: ***.:*
```

C

```
Citrus_TIR1           YDVFLSFRGEDTRDNFTSHLY-------------- 21
Populus_TIR1          YDVFLSFRGEDTRNNFTDHLYTALCQAGIHTFRDD 35
Arabidopsis_TIR1      -DVFPSFRGEDVRKTFLSHLLKEF----------- 23
                       *** ******.*..* .**

Citrus_TIR2           --------------AIEASAISVIIFSEGYASSRWCLDELVKI--- 29
Populus_TIR2          ELPRGEEISPHLWKAIQESRISIIVFSKDYASP-WCLDELVKI--- 42
Arabidopsis_TIR2      -------IGPELIQAIRESRIAIVVLSKNYASSSWCLDELVEIMKC 39
                                    **. * *:::::*:.***. *******:*

Citrus_TIR3           -----GQIVIPVFYRVDPSDVRKQTG--- 21
Populus_TIR3          CKKXTGQIVLPVFYDVDPSDVRKQTGSFA 29
Arabidopsis_TIR3      ---ELGQIVMPIFYGVDPSDVRKQ----- 21
                           ****:*:** *********

Citrus_TIR4           KENPEKVQKWRDALKEAANLSGF-- 23
Populus_TIR4          ------VQRWRDALTEAANLSGWD- 18
Arabidopsis_TIR4      ---------WRKALTDVANIAGEHS 16
                               **.**.:.**::*
```
