# Supplementary material for: Genome-Wide Comparative Analysis Reveals Similar Types of NBS Genes in Hybrid Citrus sinensis Genome and Original Citrus clementine Genome and Provides New Insights into Non-TIR NBS Genes
Source: PLoS One. 2015 Mar 26;10(3):e0121893. doi: 10.1371/journal.pone.0121893 (PMC4374887; doi:10.1371/journal.pone.0121893)
Supplement: S1 Table — (DOCX) [file pone.0121893.s012.docx]

**Table S1. The NBS genes in three Citrus genomes**

| Genome | Original NBS genes | Novel NBS genes | Total NBS genes |
| --- | --- | --- | --- |
| *Citrus clementina* | 413 | 205 | 618 |
| *Citrus sinensis* China | 499 | 151 | 650 |
| *Citrus sinensis* USA | 484 | 24 | 508 |
